# INCEPT: The Intensive Care Platform Trial—Design and protocol

**DOI:** 10.1111/aas.70023

**Published:** 2025-03-14

**Authors:** Anders Granholm, Morten Hylander Møller, Benjamin Skov Kaas‐Hansen, Aksel Karl Georg Jensen, Marie Warrer Munch, Maj‐Brit Nørregaard Kjær, Lars Wiuff Andersen, Olav Lilleholt Schjørring, Bodil Steen Rasmussen, Tine Sylvest Meyhoff, Rikke Faebo Larsen, Hans‐Christian Thorsen‐Meyer, Marie Oxenbøll Collet, Nick Frørup Meier, Stine Estrup, Ole Mathiesen, Mathias Maagaard, Lone Musaeus Poulsen, Thomas Strøm, Steffen Christensen, Camilla Rahbek Lysholm Bruun, Frederik Keus, Peter Rossing, Asger Granfeldt, Anne Craveiro Brøchner, Theis Skovsgaard Itenov, Maria Cronhjort, Jon Henrik Laake, Johanna Hästbacka, Carmen Andrea Pfortmueller, Martin Siegemund, Martin Ingi Sigurdsson, Lars Peter Kloster Andersen, Davide Placido, Theis Lange, Anders Perner

**Affiliations:** ^1^ Department of Intensive Care Copenhagen University Hospital–Rigshospitalet Copenhagen Denmark; ^2^ Section of Biostatistics, Department of Public Health University of Copenhagen Copenhagen Denmark; ^3^ Collaboration for Research in Intensive Care (CRIC) Copenhagen Denmark; ^4^ Department of Clinical Medicine, Faculty of Health Sciences University of Copenhagen Copenhagen Denmark; ^5^ Department of Clinical Medicine Aarhus University Aarhus Denmark; ^6^ Department of Anesthesiology and Intensive Care Aarhus University Hospital Aarhus Denmark; ^7^ Prehospital Emergency Medical Services, Central Region Denmark Aarhus Denmark; ^8^ Department of Anaesthesia and Intensive Care Aalborg University Hospital Aalborg Denmark; ^9^ Department of Clinical Medicine Aalborg University Aalborg Denmark; ^10^ Department of Anaesthesiology and Intensive Care Zealand University Hospital Køge Denmark; ^11^ Department of Anaesthesia and Critical Care Medicine Odense University Hospital Odense Denmark; ^12^ Department of Anaesthesia and Critical Care Medicine, Hospital Sønderjylland University Hospital of Southern Denmark Odense Denmark; ^13^ Department of Critical Care, University Medical Center Groningen University of Groningen Groningen The Netherlands; ^14^ Steno Diabetes Center Copenhagen Herlev Denmark; ^15^ Department of Anaesthesia and Intensive Care Lillebælt Hospital Kolding Denmark; ^16^ Department of Anesthesiology and Intensive Care Bispebjerg and Frederiksberg Hospitals Copenhagen Denmark; ^17^ Department of Clinical Science and Education Södersjukhuset, Karolinska Institutet Stockholm Sweden; ^18^ Department of Clinical Sciences Danderyd Hospital, Karolinska Institutet Stockholm Sweden; ^19^ Department of Anaesthesia and Intensive Care Medicine, Division of Emergencies and Critical Care, Rikshospitalet Oslo University Hospital Oslo Norway; ^20^ Department of Research and Development, Division of Emergencies and Critical Care, Rikshospitalet Oslo University Hospital Oslo Norway; ^21^ Department of Intensive Care, Tampere University Hospital Wellbeing Services County of Pirkanmaa and Tampere University Tampere Finland; ^22^ Department of Intensive Care Medicine, Inselspital University Hospital Bern Bern Switzerland; ^23^ Intensive Care Unit University Hospital Basel Basel Switzerland; ^24^ Faculty of Medicine University of Iceland Reykjavik Iceland; ^25^ Department of Anesthesiology and Critical Care Medicine Landspitali—the National University Hospital of Reykjavik Reykjavik Iceland; ^26^ Novo Nordisk Foundation Center for Protein Research University of Copenhagen Copenhagen Denmark

**Keywords:** adaptive platform trial, intensive care, randomised clinical trial, trial protocol

## Abstract

**Background:**

Adult intensive care unit (ICU) patients receive many interventions, but few are supported by high‐certainty evidence. Randomised clinical trials (RCTs) are essential for trustworthy comparisons of intervention effects, but conventional RCTs are costly, cumbersome, inflexible, and often turn out inconclusive. Adaptive platform trials may mitigate these issues and have higher probabilities of obtaining conclusive results faster and at lower costs per participant.

**Methods:**

*The Intensive Care Platform Trial* (*INCEPT*) is an investigator‐initiated, pragmatic, randomised, embedded, multifactorial, international, adaptive platform trial including adults acutely admitted to ICUs. *INCEPT* will assess comparable groups of interventions (primarily commonly used interventions with clinical uncertainty and practice variation) nested in domains. Interventions may be either open‐label or masked. New domains will continuously be added to the platform. *INCEPT* assesses multiple core outcomes selected following substantial stakeholder involvement: mortality, days alive without life support/out of hospital/free of delirium, health‐related quality of life, cognitive function, and safety outcomes. Each domain will use one of these core outcomes as the primary outcome. *INCEPT* primarily uses Bayesian statistical methods with neutral, minimally informative or sceptical priors, adjustment for important prognostic baseline variables, and calculation of absolute and relative differences in the intention‐to‐treat populations. Domains and intervention arms may be stopped for superiority/inferiority, practical equivalence, or futility according to pre‐specified adaptation rules evaluated using statistical simulation or at pre‐specified maximum sample sizes. Domains may use response‐adaptive randomisation, meaning that more participants will be allocated to interventions with higher probabilities of being superior.

**Conclusions:**

*INCEPT* provides an efficient, pragmatic, and flexible platform for comparing the effects of many interventions used in adult ICU patients. The adaptive design enables the trial to use accumulating data to improve the treatment of future participants. *INCEPT* will provide high‐certainty, conclusive evidence for many interventions, directly inform clinical practice, and thus improve patient‐important outcomes.

## 1 INTRODUCTION

The most critically ill patients are admitted to intensive care units (ICUs) where they are treated with life supportive therapies, for example, advanced respiratory and circulatory support and renal replacement therapy (RRT). Each year, approximately 25,000–30,000 patients are admitted to ICUs in Denmark.^1^ Due to demographic changes, this is expected to increase in the coming years.^2^ Approximately 1 in 5 ICU patients die within 30 days,^1^ and those who survive go through long hospital stays and recovery periods and experience decreased health‐related quality of life (HRQoL). ICUs consume vast amounts of healthcare and financial resources,^3^ and critical illness is thus a huge burden on patients, their family members, and society.^4^ Of the many interventions used in ICUs, only approximately 10% are supported by high‐certainty evidence.^5^ Concerningly, randomised clinical trials (RCTs) of ICU interventions often show either harm or lack of clinically relevant effects, and *increases* in mortality with interventions thought to be beneficial are found almost as often as *decreases*.^6^, ^7^ Consequently, multiple recent RCTs have led to de‐implementation of interventions already used clinically.^8^

While RCTs of ICU interventions already in use or new ones are warranted, there are limitations in conventional RCTs (i.e., stand‐alone, parallel‐group RCTs comparing two interventions at a time with limited flexibility, fixed sample sizes, and dichotomised conclusions based on conventional, frequentist statistical methods).^9^ Most RCTs of ICU interventions are inconclusive, as they do not show firm superiority of one intervention^6^, ^10^ and often do not formally assess practical equivalence. This is often, at least partly, explained by over‐optimistic assumed effect sizes and incorrect assumptions about baseline outcome distributions,^11^, ^12^, ^13^, ^14^, ^15^ leading to RCTs that are unable to provide firm conclusions regarding smaller, yet clinically important intervention effects. Unfortunately, inconclusive RCTs are at risk of being erroneously interpreted as if there are no differences between interventions.^12^, ^16^ Ultimately, this poses a risk of research waste and patient harm.

Adaptive platform trials^17^ may remedy some of the challenges with conventional RCTs and are receiving increased interest,^18^, ^19^ also in the ICU setting.^20^ Adaptive platform trials focus on a *population* (defined by a setting or condition) instead of specific *interventions*, with many interventions assessed on the same platform trial.^17^ Comparable interventions may be nested in so‐called *domains*,^17^ and adaptive platform trials may run perpetually with new interventions or domains added continuously.^17^ Adaptive platform trials may use frequent adaptive (interim) analyses and make multiple adaptations based on accrued data.^17^ These include early dropping of arms, stopping whenever the evidence is certain enough, and response‐adaptive randomisation, where accrued data are used to update allocation probabilities to increase chances that future participants will receive interventions that with higher probabilities will lead to better outcomes.^17^, ^21^ As adaptive platform trials typically run longer than stand‐alone trials and use the same infrastructure, improving trial infrastructure (e.g., by automating data collection) and involving stakeholders can be more feasible than in shorter‐running stand‐alone trials. By planning for the inclusion of many participants while increasing flexibility to ensure stopping as soon as the evidence is sufficiently strong, adaptive trials can be designed to have higher probabilities of obtaining conclusive answers without being *too* large, ensuring that results can benefit other patients as early as possible and that research resources are used optimally.^22^ Due to both the adaptations and the continuous use of the same trial infrastructure, adaptive platform trials may thus provide higher probabilities of conclusive answers while being more efficient and cost‐effective than multiple stand‐alone trials addressing the same questions.^17^, ^22^, ^23^ Taken together, adaptive platform trials thus convey ethical, practical, and economical benefits and may be more compelling to patients, clinicians, and other stakeholders.^9^, ^17^, ^23^, ^24^, ^25^, ^26^ This is the motivation behind the *Intensive Care Platform Trial* (*INCEPT*).

## 2 METHODS

### 2.1 Trial design and approvals

*INCEPT* is an investigator‐initiated, pragmatic, randomised, embedded, multifactorial, international, domain‐based adaptive platform trial focused on—but not restricted to—commonly used interventions where there is equipoise and practice variation due to uncertainty about which intervention is better.

*INCEPT* is governed by a *core protocol* with *domain‐specific appendices* describing specific interventions and methodological choices for each domain. The core protocol has been preceded by substantial methodological work,^27^ and extends the protocol for the adaptive Bayesian *Empirical Meropenem* *versus* *Piperacillin/Tazobactam for Adult Patients with Sepsis* (*EMPRESS*) trial.^28^

The current, first approved (version 1.3) and all future approved versions of the full core protocol and domain‐specific appendices will be available at the trial website (www.incept.dk); additional details, complete definitions, and completed reporting checklists (*Standard Protocol Items: Recommendations for Interventional Trials* [*SPIRIT*]^29^ and *Consolidated Standards Of Reporting Trials, Adaptive designs Extension* [*CONSORT‐ACE*]^30^) can be found in these documents.

### 2.2 Trial conduct, consent, and approvals

*INCEPT* will be conducted according to the approved core protocol and domain‐specific appendices and will adhere to the applicable legislation (including that for clinical trials conducted in emergency situations^31^, ^32^), the Helsinki declaration,^33^ the International Conference on Harmonization Good Clinical Practice guidelines,^34^ and the World Health Organization's guidance for best practices for clinical trials.^35^

Informed consent prior to enrolment in *INCEPT* is not possible due to acute critical illness, and thus, enrolment will generally be without prior consent,^31^, ^32^ followed by informed consent to continue in each domain (and inclusion in other domains that may become relevant during the same ICU stay) from legal surrogates and participants as soon as possible. Consent for participation may be withdrawn at any time without explanation and may be withdrawn for specific domains only. If consent is withdrawn, we will ask for permission to continue data registration.

*INCEPT* has been approved by the competent authorities and publicly registered before initiation (approvals and identifiers: EUCT number: 2024‐516208‐41‐00; ClinicalTrials.gov identifier: NCT06667999; Universal Trial Number: U1111‐1313‐8171). Additional local/national approvals will be obtained as required, and new domains added to the platform will similarly be approved before initiation.

### 2.3 Eligibility criteria

Adults (≥18 years old) acutely admitted to the ICU may be included if they are eligible for one or more active domain(s). Patients will be excluded if informed consent is expected to be unobtainable following inclusion or if they are under coercive measures. Additional specific eligibility criteria will apply for each domain. Patients may be included in *INCEPT* multiple times during separate ICU stays, but only randomised once to each domain.

### 2.4 Domains, interventions, and co‐enrolment

**FIGURE 1:**
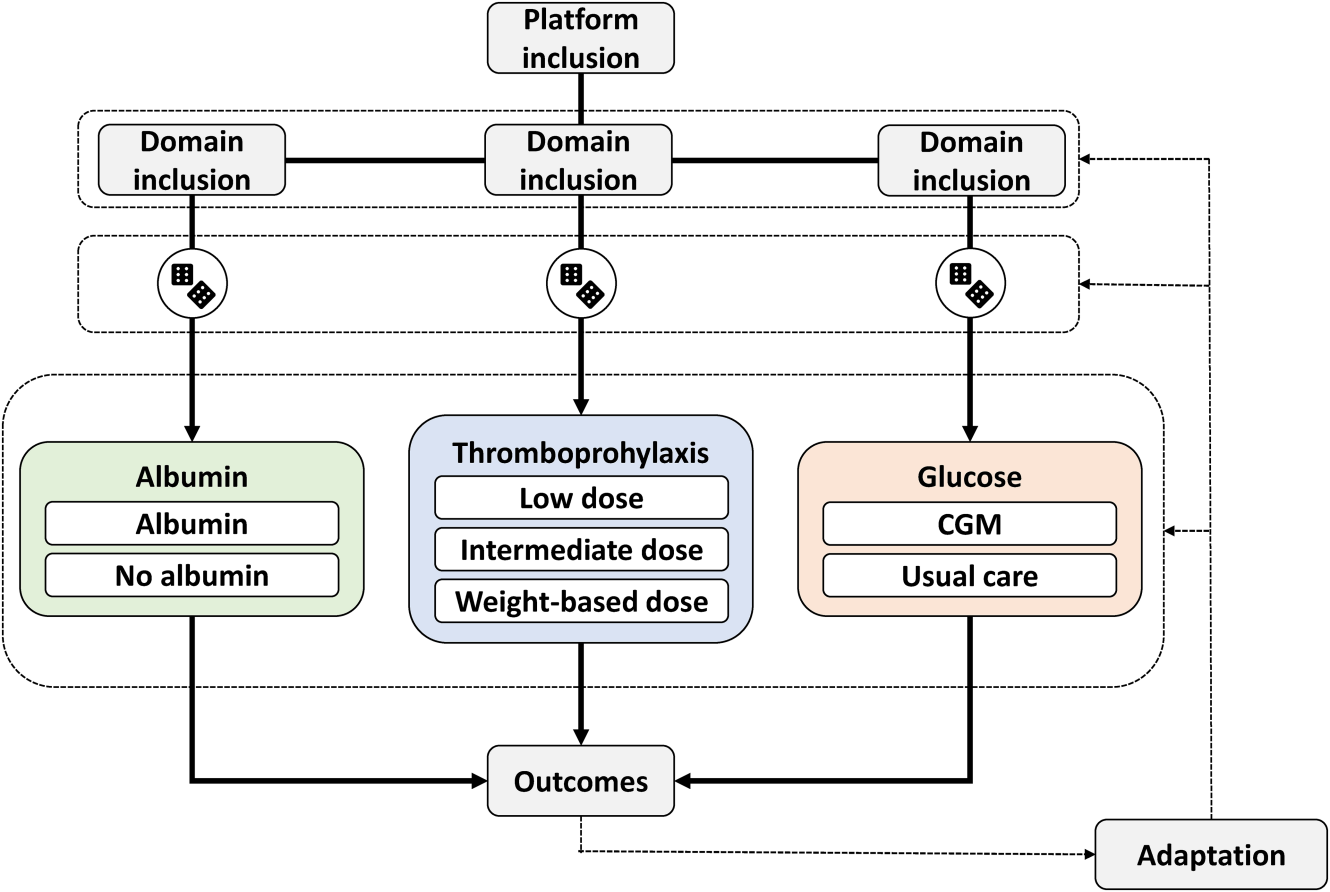
Multiple domains in an adaptive platform trial, with the domains and interventions reflecting the tentative first domains and interventions on *INCEPT*. Each domain consists of comparable interventions (similar to what could be compared in a single stand‐alone trial), and all domains use the same overall platform trial infrastructure. All domains use the same overall platform eligibility criteria, and the overall eligible population thus consists of adult, acutely admitted ICU patients. Additional domain‐specific inclusion criteria further narrow down this population, for example, the albumin domain will be restricted to patients with shock, the thromboprophylaxis domain to patients with an indication for thromboprophylaxis with LMWH, and the glucose domain to patients with an insulin requirement for blood glucose management. Randomisation to the interventions in each domain is separate. Collection of data, including outcome data, is joint across domains. Based on adaptive (interim) analyses of outcome data, domains will adapt by changing the available interventions and domains and by adapting allocation probabilities, as described in the text and full core protocol available at the trial website. Domains will be stopped once there is sufficiently strong evidence for a conclusion according to pre‐defined criteria or at a pre‐specified maximum sample size. Additional details on all *INCEPT* domains will be specified in domain‐specific appendices to the core protocol. At the time of writing, the albumin domain has been approved, while the other domains illustrated are under preparation and have not yet been submitted for approval. CGM: continuous glucose monitoring; ICU: intensive care unit; *INCEPT*: the Intensive Care Platform Trial; LMWH: low‐molecular weight heparin.

Comparable interventions will be nested in domains^17^ (Figure 1), which conceptually resemble what could be compared in a stand‐alone trial. Domains will continuously be added to the platform; however, each domain will be *closed*,^24^ that is, new interventions will not be added to existing domains once started. Interventions in domains may be open‐label or masked, that is, blinded to participants and personnel. This will be described, along with rationales, in the domain‐specific appendices. In cases where simultaneous enrolment in multiple specific domains is not considered appropriate (e.g., domains with overlapping interventions or interventions targeting the same organ systems), this will be specified along with a priority list in the domain‐specific appendices. All co‐interventions not assessed in active domains will be at the discretion of treating clinicians. Currently, the domains outlined in Figure 1 have been approved or are being planned for submission for approval later. *INCEPT* allows and prioritizes simultaneous enrolment in as many domains as possible. Co‐enrolment with other interventional trials is generally supported and permitted after approval by the management committee.

### 2.5 Outcomes

All *INCEPT* domains will assess the *core outcomes* outlined in Table 1. Additional outcomes may be assessed in specific domains. The *INCEPT* core outcomes are based on a core outcome set for adult general ICU patients developed as part of *INCEPT* and with extensive stakeholder involvement.^42^ The exact definitions and tools have been specified by the *INCEPT* management committee, as the associated *core outcome measurement set* ^42^, ^43^ has not yet been developed. For each domain, the *primary* outcome and the *guiding* outcome (the outcome used for all adaptations as described below) must be one of the core outcomes, except HRQoL, cognitive function, and domain‐specific safety outcomes. Domains will generally use the same *primary* and *guiding* outcome, but different domains may use different core outcomes as primary and guiding outcomes if relevant (e.g., the same outcome after different follow‐up durations may be used as the guiding and primary outcomes, respectively, to facilitate faster adaptations). Staff assessing HRQoL and cognitive function will always be blinded to allocation.

**TABLE 1:** INCEPT core outcomes.

Outcome	Definition and operationalization
All‐cause 30‐day mortality	All‐cause, fixed‐time mortality
All‐cause 90‐day mortality	
All‐cause 180‐day mortality	
Days alive without life support at day 30^a^	Days alive without invasive mechanical ventilation, continuous use of vasopressors/inotropes, use of RRT (continuously or intermittently, including up to 3 days between intermittent RRT) at hospitals
Days alive without life support at day 90^a^	
Days alive out of hospital at day 30^a^	Days alive and out of hospital^b^
Days alive out of hospital at day 90^a^	
Days free of delirium at day 30^a^	Days are *not* considered free of delirium in case of any of the following: (1) any registered positive delirium score with a validated screening tool^c^ (2) new treatment with antipsychotics^d^ (3) delirium not evaluable due to mortality or registered coma^e^
EQ‐5D‐5L index values^36^ (HRQoL) at day 180	EQ‐5D‐5L index values, primarily calculated using national value sets, and with non‐survivors assigned 0 (corresponding to a HRQoL as bad as being dead)^f^
EQ VAS^36^ (HRQoL) at day 180	EQ VAS with non‐survivors assigned 0 (worst possible value)^f^
Cognitive function at day 180	Assessed using the *Montreal Cognitive Assessment test 5‐minute version, v2.1* (“*Mini MoCA*”),^37^, ^38^ with non‐survivors assigned 0 (worst possible value)^f^
One or more domain‐specific safety outcomes^g^	Number of participants with one or more safety outcomes specified in the domain‐specific appendices according to a risk proportionate approach^39^ and considerations about which safety outcomes are clinically most relevant

*Note*: Core outcomes assessed in all *INCEPT* domains. Complete definitions with additional details are available in the full core protocol, along with *mock tables* illustrating how outcome data from the final and adaptive analyses will be presented.

Abbreviations: HRQoL, health‐related quality of life; *INCEPT*, the Intensive Care Platform Trial; RRT, renal replacement therapy.

^a^ Reported in two versions: non‐survivors assigned 0 (as most commonly done^41^ to ensure that mortality is the worst possible outcome) and actual values without penalizing mortality.^40^ The primary versions will be specified for each domain.

^b^ Days in rehabilitation facilities and nursing homes will not count as days in hospitals.

^c^ A list of accepted screening tools is included in the full core protocol.

^d^ Any administration of haloperidol, olanzapine, or quetiapine in participants not receiving either of these at index hospital admission.

^e^ A list of scales and cutoffs used to define coma is included in the full core protocol.

^f^ Secondary analyses will be conducted in survivors only.

^g^ The maximum follow‐up period must cover at least the interventional period; for domains using a primary/guiding outcome with a shorter follow‐up duration than the maximum interventional period, this outcome will additionally be reported after a follow‐up duration corresponding to the follow‐up duration for the primary/guiding outcome.

### 2.6 Randomisation and allocation concealment

Randomisation will be separate and independent in each domain via an electronic system ensuring concealed allocation. Domains may use either fixed allocation probabilities, response‐adaptive randomisation (meaning that participants will have higher probabilities of being allocated to interventions with higher probabilities of being superior^21^), or combinations (e.g., a fixed allocation probability to a common control arm, if any, and response‐adaptive randomisation in non‐control arms).^21^ When response‐adaptive randomisation is used, allocation probabilities will be updated after adaptive analyses according to the probabilities of each arm being overall superior; response‐adaptive randomisation may be restricted to avoid over‐aggressive adaptations to random fluctuations (Section 2.12).^21^, ^44^

Domains using response‐adaptive randomisation will generally use block randomisation stratified by site (and possibly additional anticipated important baseline prognostic factors) prior to the first adaptive analysis, followed by simple, unstratified randomisation.^45^ Domains using fixed randomisation only will generally use block randomisation stratified by site (and possibly additional baseline variables) throughout the enrolment.

### 2.7 Baseline data, process data, separation, and feasibility

The baseline characteristics outlined in Table 2 will be registered and presented for all domains; in addition, domains may register and present additional relevant baseline data. Relevant process data (including data on protocol adherence) will be registered in each domain and may be used to assess separation between arms where relevant. Domains may use formal feasibility phases with pre‐defined feasibility criteria.

**TABLE 2:** Baseline variables.

Characteristic
Country of enrolment
*[Each participating country will be listed]*
Age (years)
Sex
Female
Male
Weight (kg)
Height (m)
Use of invasive mechanical ventilation
Use of vasopressors/inotropes
Use of RRT
Limitations of care
Co‐existing conditions
Active haematological malignancy or metastatic cancer
History of ischaemic heart disease or heart failure
Diabetes mellitus
Chronic pulmonary disease
Chronic liver disease
Known use of immunosuppressive therapy within the last 3 months
Previous organ transplantation
Chronic use of RRT
Treatment with antipsychotics at hospital admission
Acute surgery within 7 days prior to randomisation
SMS‐ICU^a^
Clinical Frailty Scale^b^
Lowest systolic blood pressure in the 24 h preceding randomisation (mmHg)
Highest plasma lactate in the 24 h prior to randomisation (mmol/L)
Highest plasma creatinine in the 24 h prior to randomisation (μmol/L)

*Note*: Baseline data assessed in all *INCEPT* domains. Variables are binary unless stated otherwise. Complete definitions with additional details are available in the full core protocol, along with a generic *mock table* illustrating how baseline data will be presented.

Abbreviations: mmHg, millimetres of mercury; mmol/L, millimoles per litre; RRT, renal replacement therapy; SMS‐ICU, Simplified Mortality Score for the Intensive Care Unit^46^; μmol/L, micromoles per litre.

^a^ SMS‐ICU^46^ is a severity score ranging from 0 to 42 points, with higher scores indicating more severe illness and higher risks of death.

^b^ Investigator‐assessed clinical frailty, using the Clinical Frailty Scale v2.0.^47^, ^48^ Values range from 1 (very fit) to 9 (terminally ill).

### 2.8 Statistical framework and general statistical considerations

*INCEPT* will primarily use Bayesian statistical methods^49^ for adaptive and final analyses, as this approach is well‐suited and commonly used for advanced adaptive trials^9^, ^17^, ^18^, ^50^ and has interpretational advantages.^49^ Specific domains may use conventional, frequentist statistical methods with no or few adaptations. Members of the statistical analysis team will be unblinded and will not participate in screening, inclusion, intervention delivery, or outcome assessment to avoid any potential influence on decisions regarding enrolments, treatment, or outcome assessment.^51^ In addition, statistical analysis plans will be very detailed, and analysis scripts will be prepared using simulated data prior to initiating domains. The primary conclusions in each domain will be based on whether a stopping rule (Section 2.11) is ultimately triggered, and interpretations will in any case be probabilistic.

### 2.9 Analysis sets, comparisons, and estimands

Analyses will primarily be conducted in full analysis sets according to the intention‐to‐treat principle, that is, all randomised participants except those without consent to the use of *any* data (except if specified otherwise, with motivations, for specific domains). Patients erroneously randomised to blinded domains may be excluded if they have not received the allocated intervention and are excluded before unblinding and analysis.^52^ Additional analyses may be conducted using other analysis sets (e.g., per‐protocol populations).

In domains with >2 arms, arms may either be compared simultaneously *all* versus *all* or pairwise against a *common control arm*.^17^, ^21^ Domains using a control arm (i.e., an arm corresponding to usual care or placebo) may be analysed using either strategy, generally informed by statistical simulation.^44^ Participants will only be compared with participants randomised in the same domain. As *INCEPT* domains are *closed* and *staggered entry* is not permitted, participants will thus only be compared with *concurrently* randomised participants, thereby avoiding potential biases due to the use of non‐concurrent controls.^53^

Key attributes (population, interventions, outcome, summary measure, and handling of intercurrent events) for the primary estimand in each domain,^54^, ^55^ and thus the questions primarily answered will be clearly specified in domain‐specific appendices.

### 2.10 Adaptive analyses

The timing and frequency of adaptive (interim) analyses will be specified for each domain. Adaptive analyses will be conducted at specified times (e.g., the first workday of the month) whenever a pre‐specified number of participants has completed their outcome‐data lag period,^56^ defined as the follow‐up duration of the guiding outcome plus a 15‐day data collection/verification period. Adaptive analyses will include all participants who have completed their outcome‐data lag period; that is, participants with outcome data available who have not completed the period will not be analysed.

### 2.11 Adaptation rules

Most *INCEPT* domains will use stopping rules based on Bayesian probabilities as outlined in this section, but other stopping rules (including, e.g., conventional group sequential monitoring boundaries^57^) may be used in specific domains.

Domains may be stopped, and arms may be dropped, for either inferiority/superiority (mandatory), practical equivalence (optional), or futility (optional, only in domains with comparisons against a *common control arm*),^21^ based on the posterior probabilities for analyses of the *guiding* outcome and pre‐defined thresholds, with all stopping rules being binding.^51^

#### 2.11.1 Adaptation rules in domains simultaneously comparing all arms

For domains simultaneously comparing all arms, arms will be dropped for inferiority if the posterior probability that an arm is overall best is below a pre‐specified threshold.^21^, ^44^ Similarly, if the probability that a single arm is overall best exceeds a pre‐specified threshold, the domain will be stopped and that arm declared superior.^21^, ^44^ Domains may be stopped for practical equivalence if the absolute difference between all remaining active arms is less than a pre‐defined size according to a pre‐defined probability threshold.^21^, ^44^

#### 2.11.2 Adaptation rules in domains with pairwise comparisons against a common control

For domains with pairwise comparisons of interventional arms against a common control arm, non‐control arms will be dropped for inferiority if the probability that they are better than the common control is below a pre‐specified threshold.^21^, ^44^ If the probability that a non‐control arm is superior to the common control arm exceeds a pre‐specified threshold, the common control arm will be dropped, and the superior non‐control arm promoted to be the new control (if multiple non‐control arms are superior in the same analysis, the one with the highest probability of being overall superior will become the new control).^17^, ^21^, ^44^ A domain will be stopped for superiority when only one arm remains after dropping the last comparator for inferiority. In domains with a common control arm, non‐control arms may be dropped for practical equivalence if the absolute difference compared to the common control arm is less than a pre‐defined size according to a pre‐defined probability threshold. Similarly, non‐control arms may be dropped for futility if they are not superior by a pre‐specified amount compared to the common control arm according to a pre‐specified probability threshold.^21^, ^44^ Assessments of practical equivalence and/or futility may be conducted against the original common control arm only or also against other arm(s) promoted to be the common control arm.

#### 2.11.3 Other considerations

Whenever arms are dropped in domains without a common control arm or whenever a common control arm is replaced, analyses will be immediately repeated, as this will lead to updated probabilities for the remaining comparisons that in turn may lead to stopping rules being triggered for additional arms. Domains will be declared inconclusive if they reach the maximum sample size without triggering applicable stopping rules. Whenever domains are stopped, all remaining randomised participants will be followed up, and a final analysis conducted. The primary conclusion (superiority/inferiority, practical equivalence, futility, or inconclusiveness) will be based on the last adaptive analysis, while the primary estimates and probabilities presented will be from the final analysis, including data from all randomised participants.

Stopping rules for superiority and inferiority will generally be calibrated using statistical simulation^9^, ^21^, ^44^ to obtain an overall type 1 error rate for the guiding outcome of ≤5% (i.e., there will be ≤5% probability of stopping for superiority if there are no between‐arm differences).

### 2.12 Randomisation rules

Domains may use either fixed allocation profiles (with appropriate rescaling if arms are dropped), response‐adaptive randomisation,^21^, ^57^ or combinations hereof. For domains with two arms or all arms compared simultaneously, we will generally use initial equal allocation followed by either fixed equal allocation or response‐adaptive randomisation. For domains comparing interventional arms against a common control arm, we will generally use a fixed and relatively higher control arm allocation probability (e.g., square‐root to number of non‐control arms to 1‐ratios^21^), and either response‐adaptive randomisation or fixed allocation probabilities in the remaining arms. When response‐adaptive randomisation is used, this will be based on the probabilities of each arm being overall superior,^21^, ^44^, ^58^ and restrictions (minimum/maximum limits on probabilities and/or use of a *softening factor* making allocation probabilities more similar than the raw probabilities of each arm being superior^21^, ^44^) may be used to avoid over‐aggressive adaptations to random fluctuations and mitigate the potential undesirable effects on performance metrics that may be seen in some cases. Exact randomisation rules will be specified separately for each domain.

### 2.13 Analyses

#### 2.13.1 Descriptive data

Baseline, process, and outcome data will be presented descriptively in each arm in each domain. Numerical data will be summarized using medians with interquartile ranges (IQRs) and categorical variables as counts with percentages.

#### 2.13.2 Analysis models, priors, and presentation of results

Analyses will generally use Bayesian logistic and linear regression models adjusted for the stratification variables used and several additional anticipated important prognostic baseline variables to increase precision and account for potential baseline imbalances^59^: site, age, sex, active hematologic malignancy or metastatic cancer, acute surgery within 7 days prior to randomization, invasive mechanical ventilation, circulatory support (continuous infusion of vasopressors/inotropes), RRT within 72 h prior to randomization, and time period (categorical; used in all domains using arm dropping or response‐adaptive randomisation, with a new period defined following each adaptation). Continuous variables will be modeled using linear and quadratic terms on the model scale to allow for non‐linearity.

We will generally use neutral priors (i.e., priors not favouring any intervention) for the intervention effects and all adjustment variables in the primary analyses. Priors for the intervention effects will generally be weakly informative or slightly to moderately sceptical, which limits the influence of random fluctuations early.^60^ Sensitivity analyses using other priors (e.g., more informative/sceptical priors, evidence‐based priors, or expert‐elicited priors^49^, ^61^) will generally be conducted. Exact priors will be specified in each domain‐specific appendix.

Results will be presented as sample‐average (marginal) absolute differences (risk differences/mean differences; primary summary measures) and relative differences (risk ratios/ratios of means; secondary summary measures) using a robust G‐computation‐based approach.^59^ Posteriors will be summarised using median values as point estimates, with 95% percentile‐based credible intervals and graphical presentation of full posterior distributions for the absolute and relative differences and the expected probabilities or mean values with each intervention. Probabilities of superiority/inferiority of all arms (overall and/or pairwise/compared to a common control) will be presented, as will all probabilities relevant for any stopping rules for practical equivalence and/or futility.

Model fitting and assessment of model adequacy are described in the full protocol. Relevant sensitivity analyses will be pre‐specified in each domain‐specific appendix or conducted post hoc if relevant.

#### 2.13.3 Heterogeneous intervention effects and interactions

The primary and adaptive analyses in *INCEPT* focus on average intervention effects, which drive all adaptations. However, potential heterogeneous intervention effects^9^, ^62^ will generally be assessed once domains close; exact plans for assessing heterogeneous intervention effects (i.e., baseline characteristics considered and the exact methodological approach) will be pre‐specified for each domain. Similarly, where deemed relevant, we will pre‐specify plans for assessing potential between‐domain interactions.

#### 2.13.4 Missing data handling

Missingness is expected to be limited for most variables and outcomes (except HRQoL and cognitive function, where moderate amounts are expected^41^) due to the variables selected and continuous monitoring of missingness in the electronic case report form (eCRF). Total and intervention‐specific missingness will be presented, and all analyses will be conducted after multiple imputation of missing data.^63^ We will assume that data are missing at random and impute 25 datasets separately in each intervention group using the predictive mean matching method for numerical variables and binary/ordinal/polytomous logistic regression methods for binary/ordinal/nominal variables, respectively.^63^ All variables included in the analyses and relevant additional baseline data (specified in the domain‐specific appendices) will be included in the imputation models for the final analyses; the imputation models for the adaptive analyses will be simplified and will include all adjustment variables, the guiding outcome, and mortality after the same follow‐up as the guiding outcome. Partially available data will be used to the extent possible. Supplementary best‐worst/worst‐best case sensitivity analyses^64^ may be used, as defined in the domain‐specific appendices.

#### 2.13.5 Statistical simulation and performance metrics

Domain designs will be evaluated with statistical simulations using the *adaptr* ^65^ R package (inceptdk.github.io/adaptr), developed as part of *INCEPT*, and according to the principles described elsewhere.^21^, ^44^ In brief, performance metrics of the final design and several variations will be evaluated under multiple clinical scenarios (always including a scenario with no differences between arms and multiple scenarios with between‐arm differences present, for example, small and large differences) using 100,000 simulations under each scenario for the final design.^21^, ^44^ The influence of key assumptions underlying the simulations (e.g., the refence distribution of the guiding outcome and the expected inclusion rates) will be assessed in sensitivity analyses.^44^

Multiple performance metrics, including expected sample sizes, will be calculated to cover both practical/logistical/economical aspects, benefits to participants and external patients, and accuracy, and presented for the final design and the evaluated sensitivity analyses.^21^, ^44^ As a general principle, domains in *INCEPT* will be required to have at least a 90% probability of conclusiveness, that is, of triggering a stopping rule at or before the maximum permitted sample size for the final design across all clinical scenarios (evaluated according to the primary assumptions regarding the reference distribution and expected inclusion rates^44^).

### 2.14 Stakeholder involvement

Involvement of key stakeholders, including former patients and members of the public, is central in *INCEPT*. To ensure the relevancy of *INCEPT* to those who will ultimately be affected by or use its results, involvement throughout the various research stages is prioritized as recommended.^35^, ^66^ Stakeholder involvement is ensured through a central advisory board to *INCEPT* and multiple local research panels. A description of stakeholder involvement so far, along with a description of the minimum requirements for stakeholder involvement in *INCEPT* going forward, is presented in the full core protocol.

### 2.15 Data collection and embedding

*INCEPT* will use a customised eCRF for inclusion, randomisation, and data collection. The eCRF will support hybrid data entry, that is, manual data entry and automated data collection from electronic patient records. Automated data collection will be expanded over time to support additional data points and electronic patient records, reducing the resource burden from manual data entry. We aim to later build embeddings within electronic patient records to allow automatic notifications to clinicians and research staff regarding eligible patients, outcomes (including safety outcomes), and more.

### 2.16 Organisational aspects, independent data monitoring and safety committees, and monitoring

*INCEPT* is governed by a platform management committee led by the platform sponsor, with the involvement of representatives from the partnering organisations/regions/countries and active domains. The platform management committee receives advice or obtains input from the competent authorities, funders, and key stakeholders via the advisory board and research panels or directly. Daily management of each domain is the responsibility of a domain management committee, chaired by a domain sponsor, that refers to the overall platform management committee.

Each domain has its own multidisciplinary independent data monitoring and safety committee (IDMSC), responsible for overseeing domain conduct and participant safety, including during annual safety meetings. IDMSCs will be advisory to the domain/platform management committees, and an IDMSC charter will be outlined for each domain. Of note, IDMSCs will not conduct the regular adaptive analyses, but will be updated about their results.

*INCEPT* and all domains will be externally monitored according to the *Good Clinical Practice* directive, with monitoring plans developed in collaboration with the *GCP unit* of *Copenhagen University Hospital*. In addition, trial and domain progress will be centrally monitored to optimize data quality and protocol adherence.

### 2.17 Dissemination

All results of all domains will be published in international peer‐reviewed medical journals, the applicable trial registers, and the trial website, with domain reports following the *CONSORT‐ACE* guideline.^30^ Generally, we aim to make results public on pre‐print servers simultaneously with submission for publication, although we may deviate from this in some instances.

## 3 DISCUSSION

*INCEPT* is an investigator‐initiated, pragmatic, randomised, embedded, multifactorial, international, domain‐based (primarily) Bayesian adaptive platform trial including adults acutely admitted to the ICU. *INCEPT* may run perpetually and intends to assess many interventions, primarily focusing on interventions in common use where there is practice variation due to a lack of high‐certainty evidence about which intervention is better.

### 3.1 Strengths

*INCEPT* comes with multiple strengths. First, it will be conducted in many ICUs across many countries, increasing the total number of eligible patients and ensuring generalizability and external validity. Currently, inclusion is planned in Denmark, the Netherlands, Sweden, Norway, Switzerland, Finland, and Iceland. Second, by being a potentially perpetual platform trial, *INCEPT* makes the establishment of a lasting infrastructure and *embedding* in both clinical practice and within electronic patient records feasible, which will facilitate (partially) automated data collection, decreasing the labor and cost per randomized participant. Third, *INCEPT* will be pragmatic, with co‐interventions administered at the discretion of the treating clinicians, and with support for co‐enrollment with other RCTs. Fourth, combining large maximum sample sizes with adaptive features will ensure high probabilities of obtaining conclusive results to directly inform clinical practice without risking that domains become *too* large. In addition, adaptive arm dropping and response‐adaptive randomization (where used) allow domains to *continuously learn* from the accumulating evidence *before* a stopping decision is triggered, which is central to a *learning healthcare system*.^67^ Adaptive arm dropping increases efficiency by dropping inferior (or equivalent or futile) arms early. Whenever inferior arms are dropped early, the probabilities of better outcomes for future participants are increased.^17^, ^21^ Similarly, response‐adaptive randomization increases the probabilities that future participants will be allocated to superior interventions.^17^, ^21^ Fifth, *INCEPT* primarily uses Bayesian statistical methods, and results will be interpreted probabilistically and nuanced regardless of whether any formal stopping rules are triggered, to embrace any uncertainties without misinterpretation. Sixth, *INCEPT* is based on a comprehensive and thorough framework for planning domains, with the central design features and assumptions evaluated using extensive statistical simulation.^21^, ^44^ This ensures an optimal trade‐off between the pros and cons of various design features, for example, the benefits of adaptive features for individual participants and external patients, and the efficiency of each domain will be carefully evaluated, and potentially negative implications of overly aggressive adaptations will be avoided. Seventh, extensive stakeholder involvement in *INCEPT* ensures the relevancy of trial results to patients, family members, clinicians, researchers, and other key stakeholders. Importantly, all domains will assess highly patient‐important core outcomes selected through comprehensive stakeholder involvement,^42^ and the guiding/primary outcome in each domain will always be one of these core outcomes.

### 3.2 Limitations

*INCEPT* comes with limitations, too. First, as maximum sample sizes in most domains are expected to be substantially larger than the *expected* sample sizes^21^, ^44^ and often larger than typical ICU RCTs,^68^ the planning and funding of domains is more complex than for most typical stand‐alone trials. This is, however, unavoidable to ensure high probabilities of conclusiveness, so results can ultimately inform clinical practice. Second, to benefit from being a platform trial, more standardisation between domains is required than in comparable stand‐alone trials. Consequently, some compromises compared to the ideal stand‐alone trial may be necessary in *INCEPT* domains. Importantly, *INCEPT* allows and encourages co‐enrolment with other trials. As such, stand‐alone trials can still be conducted alongside *INCEPT* for research questions where the compromises necessary would be deemed too large for a research question to be answered optimally within *INCEPT*. Third, the use of adaptive features increases complexity, and all adaptive features may not always be beneficial.^21^, ^57^, ^58^ By evaluating key methodological decisions in domains through extensive statistical simulation,^44^ we will ensure that the added complexity of all adaptive choices measures up to their benefits and that adaptations are only used to the extent they are deemed overall advantageous. Finally, *time drift* ^9^, ^17^, ^53^—potential bias due to changes over time in allocation probabilities and in the included populations or concomitant care—may pose a challenge and introduce bias in advanced adaptive trials if not adequately handled. Consequently, *INCEPT* adjusts all analyses for time period and only includes concurrent controls in all comparisons.

## 4 CONCLUSIONS

*INCEPT* provides an efficient and flexible platform for comparing the effects of many interventions used in adult ICU patients. The perpetual nature of the platform facilitates the establishment of lasting, improved trial infrastructure, including *embedding* the trial in both clinical practice and electronic patient records to enable (partially) automated data collection to reduce labor and cost. The use of large maximum sample sizes and advanced adaptive features ensures high probabilities of conclusiveness and enables the trial to use accumulating data to improve the treatment of future participants. The pragmatic design and advanced adaptive features mean that *INCEPT* will provide high‐certainty, conclusive evidence for many interventions. *INCEPT* will thus enable the assessment of many interventions in critically ill adult ICU patients and will provide conclusive evidence that can directly inform practice and improve patient‐important outcomes.

## AUTHOR CONTRIBUTIONS

**Conceptualisation**: AG, MHM, TL, AP. **Funding acquisition**: AG, MHM, TSM, FK, PR, TL, AP. Investigation: all authors. **Methodology**: All authors. **Project administration**: AG, MHM, BSKH, MBNK, TSM, RFL, AP. **Software**: AG, BSKH, AKGJ, TL. **Supervision**: MHM, AKGJ, TL, AP. Visualization: AG. **Writing—original draft**: AG: **Writing—review and editing**: All authors.

## FUNDING INFORMATION

*INCEPT* is funded by the *Novo Nordisk Foundation* and *Sygeforsikringen ‘danmark’*, with additional support from *Grosserer Jakob Ehrenreich og Hustru Grete Ehrenreichs Fond*, *Dagmar Marshalls Fond*, and *Savværksejer Jeppe Juhl og hustru Ovita Juhls Mindelegat*. The *INCEPT‐Albumin* domain is partially funded by *Danmarks Frie Forskningsfond*. None of the funders have had any influence on the planning of the platform trial, and none will have any influence on the design, conduct, analysis, or reporting of any domains assessed on the platform. None of the funders will have ownership of any trial data.

## CONFLICT OF INTEREST STATEMENT

Bodil Steen Rasmussen: unrestricted grant from the Novo Nordisk Foundation for long‐term follow‐up in the Handling Oxygenation Targets in the Intensive Care Unit (HOT‐ICU) trial (ended June 2024). Tine Sylvest Meyhoff: coordinating investigator of the CLASSIC trial (NCT03668236) which was supported by a grant (NNF17OC0028608) from the Novo Nordisk Foundation and by the Sofus Friis' Foundation, Rigshospitalet's Research Council, and supported by the Danish Society of Anesthesiology and Intensive Care Medicine. Hans‐Christian Thorsen‐Meyer: research grant from the Novo Nordisk Foundation (NNF19OC0054863). Marie Oxenbøll Collet: the Novo Nordisk Foundation, Grant NNF21OC0072048 Postdoc fellowships in Nursing Research 2021. Ole Mathiesen: funding to research group from the Novo Nordisk Foundation and Sygeforsikringen ‘danmark’ for other projects. Mathias Maagaard: holds shares in Novo Nordisk. Peter Rossing: grants for investigator‐initiated studies to Steno Diabetes Center Copenhagen, from Novo Nordisk, Bayer, AstraZeneca, and Lexicon. Honoraria to Steno Diabetes Center Copenhagen for steering group membership, consultancy, and education from AstraZeneca, Abbott, Bayer, Boehringer Ingelheim, Eli Lilly, Novo Nordisk, Gilead, and Sanofi. No personal honoraria and no shares/patents. Asger Granfeldt: paid member of data safety monitoring board for Noorik Pharmaceuticals (ended November 2022), work for hire for NMD Pharma, and task force member of the International Liaison Committee on Resuscitation (ILCOR) advanced life support task force. Theis Skovsgaard Itenov: the Department of Anaesthesiology and Intensive Care, Copenhagen University Hospital—Bispebjerg and Frederiksberg Hospital has a collaboration with Radiometer Medical Denmark on the testing and evaluation of equipment. The department receives reimbursements from the company. No individual receives any personal benefits or payments from the collaboration. Johanna Hästbacka: advisory board honorary fee from Paion (2022). Carmen Andrea Pfortmueller: no personal conflict of interests (personal financial interest). The department of Intensive Care at Inselspital, University Hospital of Bern reports grants from Orion Pharma, Abbott Nutrition International, B. Braun Medical AG, CSEM AG, Edwards Lifesciences Services GmbH, Kenta Biotech Ltd., Maquet Critical Care AB, Omnicare Clinical Research AG, Nestle, Pierre Fabre Pharma AG, Pfizer, Bard Medica S.A., Abbott AG, Anandic Medical Systems, Pan Gas AG Healthcare, Bracco, Hamilton Medical AG, Fresenius Kabi, Getinge Group Maquet AG, Dräger AG, Teleflex Medical GmbH, Glaxo Smith Kline, Merck Sharp and Dohme AG, Eli Lilly and Company, Baxter, Astellas, Astra Zeneca, CSL Behring, Novartis, Covidien, and Nycomed outside the submitted work. The money was paid into departmental funds; no personal financial gain applied. Davide Placido: holds stocks in Novo Nordisk. Theis Lange: served on data safety monitoring boards in studies run by Novo Nordisk and Leo Pharma. Anders Perner: research grants from the Novo Nordisk Foundation. All other authors: no relevant conflicts of interest.

## Data Availability

No patient data were analysed in this protocol manuscript. Data sharing plans for the trial are described in the full protocol available at the trial website.
